# Medication guide for dose adjustment and management of cardiotoxicity and lipid metabolic adverse events of oral antineoplastic therapy

**DOI:** 10.3389/fonc.2023.1220305

**Published:** 2023-08-25

**Authors:** Elena Ramos-Ruperez, Vicente Escudero-Vilaplana, Paula Ruiz-Briones, Roberto Collado-Borrell, Cristina Villanueva-Bueno, José Luis Revuelta-Herrero, Eva González-Haba, Xandra Garcia-Gonzalez, Sara Ibañez-Garcia, Sara Perez-Ramirez, Eduardo Zatarain-Nicolás, Ana Herranz, María Sanjurjo

**Affiliations:** ^1^ San Pablo Centro de Estudios Universitarios (CEU), University, Madrid, Spain; ^2^ Pharmacy Department, Hospital General Universitario Gregorio Marañón, Madrid, Spain; ^3^ Instituto de Investigación Sanitaria Gregorio Marañón, Madrid, Spain; ^4^ Medical Oncology Department, Hospital General Universitario Gregorio Marañón, Madrid, Spain; ^5^ Cardiology Department, Hospital General Universitario Gregorio Marañón, Madrid, Spain; ^6^ Centro de Investigación Biomédica en Red de Enfermedades Cardiovasculares (CIBER-CV), Madrid, Spain; ^7^ Complutense University, Madrid, Spain

**Keywords:** adverse event, cancer, cardiology, cardiotoxicity, oral antineoplastic therapy, safety

## Abstract

**Objective:**

The management of cardiotoxicity concerning the use of oral antineoplastic agents (OAAs) is a challenge for healthcare professionals. Our objective was to create a comprehensive medication management guide with dose adjustment recommendations on OAAs concerning cardiotoxic and lipid metabolic adverse events (AEs) to assist healthcare professionals when prescribing OAAs.

**Materials and methods:**

A review of the available information on all dose adjustments necessary to safely prescribe and dispense OAAs concerning cardiotoxicity was conducted. In January 2023, we identified all OAAs authorized by the European Medicines Agency (EMA). For each drug, the latest summary of product characteristics (SPC) approved by the EMA and the tertiary data source Lexicomp^®^ were reviewed. Cardiotoxic AEs were recorded, namely, QT interval prolongation, decrease in left ventricular ejection fraction (LVEF), imbalances in blood pressure (hypertension and hypotension), alterations in heart rate (tachycardia and bradycardia), and thrombosis. Any available dose adjustment recommendations in case of an occurrence of these adverse events were collected.

**Results:**

In all, 93 different OAAs had been approved by the EMA and were reviewed. Among them, 51.6% have recognized cardiotoxic AEs and 10.8% can cause alterations in lipid metabolism. A total of 27 (29.0%) OAAs had specific recommendations regarding QT prolongation; 88.9% were listed in the SPC and 59.3% in Lexicomp^®^. Eight OAAs (9.68%) have reported a decrease in LVEF, and four of these drugs, namely, encorafenib, lorlatinib, ripretinib, and sunitinib, have specific management recommendations. Almost half (49.5%) of currently approved OAAs can potentially alter blood pressure; 34 (36.6%) of them have been reported to cause hypertension and 12 (12.9%) are related to hypotension. Tachycardia and/or bradycardia are associated with 22.6% and 8.6% of the evaluated drugs, respectively. Regarding thrombosis, 30 (32.3%) of the drugs analyzed included the appearance of a thrombus as a possible AE.

**Conclusions:**

More than half of the OAAs can produce cardiotoxic effects, with the most frequent being blood pressure alteration and QT interval prolongation with a non-depreciable incidence of LV dysfunction or thrombosis. Before starting the treatment, it is necessary to stratify baseline cardiovascular risk, plan a surveillance schedule, and consider referral to cardio-oncology units.

## 1 Introduction

Oncohematology is the medical area with the largest pipeline of molecules in research. Oral antineoplastic agents (OAAs) currently represent a large percentage of these new treatments. Most OAAs act specifically against specific molecular targets, increasing their effectiveness. However, OAAs have a narrow therapeutic range, making them high-risk drugs that, without proper dosing and follow-up, have severe consequences (1).

OAAs present class adverse effects (AEs) that can lead to treatment interruption or, in the most severe cases, to the patient’s death. The most frequent AEs related to OAAs are gastrointestinal, hematological, and skin effects (2). The patients’ age, polypharmacy, and the presence of other comorbidities make patients especially vulnerable to drug AEs (3). A common comorbidity is cardiovascular (CV) disease, with approximately 11.3% of cancer patients having a previous cardiac history (4), including left ventricular dysfunction, atrial fibrillation, hypertension, and QT prolongation/arrhythmias.

Despite increased recognition and consensus of cardiotoxicity from cancer therapy (5) and recent international guidelines of cardio-oncology, such as the European Society of Cardiology (ESC) Guidelines (6), clinical care for patients remains a challenge due to the variability of these undesirable effects among the different OAAs, the high number of new molecules, and the low frequency of some of the toxicities. Besides, it should be noted that clinical trials exclude patients in risk situations, such as those with previous CV pathologies, making recommendations on these drugs challenging to make in many cases (6). Thus, due to the rapid development and availability of new OAA drugs, it is crucial to propose an updated practical approach to monitor and manage CV toxicities to improve the clinical response, medication safety, and quality of life of cancer patients treated with OAAs.

Our objective was to create a comprehensive medication management guide with dose adjustment recommendations on OAAs concerning cardiotoxic and lipid metabolic AEs to assist healthcare professionals in oncology and hematology when prescribing these drugs.

## 2 Materials and methods

A review of the available information on all dose adjustments necessary to safely prescribe and dispense OAAs concerning cardiotoxicity was conducted.

In January 2023, we identified all OAAs authorized by the European Medicines Agency (EMA). We considered as OAAs those drugs for oral administration divided into the following groups of drugs according to their Anatomical Therapeutic Chemical (ATC) (7): plant alkaloids (etoposide, topotecan, and vinorelbine), alkylating agents (busulfan, cyclophosphamide, chlorambucil, lomustine, and melphalan), anaplastic lymphoma kinase (ALK) inhibitors (alectinib, brigatinib crizotinib, ceritinib, and lorlatinib), antimetabolites (azacitidine, capecitabine, methotrexate, mercaptopurine, and trifluridine/tipiracil), BCR-ABL tyrosine kinase inhibitors (asciminib, bosutinib, dasatinib, imatinib, nilotinib, and ponatinib), Bruton’s tyrosine kinase (BTK) inhibitors (acalabrutinib, ibrutinib, and zanubrutinib), cyclin-dependent kinase (CDK) inhibitors (abemaciclib, palbociclib, and ribociclib), epidermal growth factor receptor (EGFR) tyrosine kinase inhibitors (afatinib, erlotinib, gefitinib, osimertinib, and poziotinib), hormone antagonists and related agents (abiraterone, anastrozole, apalutamide, bicalutamide, daralutamide, enzalutamide, exemestane, letrozole, relugolix, and tamoxifen), human epidermal growth factor receptor 2 tyrosine kinase receptor 2 (HER2) inhibitors (lapatinib, neratinib, and tucatinib), immunosuppresants (lenalidomide, pomalidomide, and thalidomide), Janus kinase (JAK) inhibitors (ferdatinib and ruxolitinib), mammalian target of rapamycin (mTOR) inhibitors (everolimus), mitogen-activated protein kinase (MEK) inhibitors (binimetinib, cobimetinib, and trametinib), polyadenosine diphosphate [ADP]–ribose polymerase (PARP) inhibitors (niraparib, olaparib, and rucaparib), serine/threonine kinase BRAF inhibitors (encorafenib, dabrafenib, and vemurafenib), vascular endothelial growth factor tyrosine kinase receptor (VEGF) inhibitors (axitinib and tivozanib), other protein kinase inhibitors (avapritinib, cabozantinib, capmatinib, gliteritinib, lenvatinib, midostaurine, nintedanib, pazopanib, pralsetinib, regorafenib, ripretinib, selpercatinib, sorafenib, sunitinib, tepotinib, and vandetanib), FGFR2 inhibitors (pemigatinib), phosphatidylinositol-3-kinase (Pi3K) inhibitors (alpelisib, idelalisib, and duvelisib), and other antineoplastic agents (hydroxycarbamide, ixazomib, selinexor, sonidegib, sotorasib, temozolomide, tretinoin, vismodegib, and venetoclax).

For each drug, the latest summary of product characteristics (SPC) approved by the EMA was reviewed. In cases where different prescription drugs with the same active ingredient were available (i.e., generic drugs), the SPC for the originally registered product was consulted. Besides, we reviewed the information available in the toxicity section for each different OAA at the tertiary data source Lexicomp^®^. Adverse events considered as cardiotoxicity and gathered for each OAA from both data sources were included, namely, QT interval prolongation, decrease in left ventricular ejection fraction (LVEF), imbalances in blood pressure (hypertension and hypotension), alterations in heart rate (tachycardia and bradycardia), and thrombosis. Due to their relationship with cardiovascular risk, alterations in lipid metabolism (hypertriglyceridemia and hypercholesterolemia) were also evaluated. Any available dose adjustment recommendations in case of an occurrence of these adverse events were collected.

The severity of each AE was graded according to the Common Terminology Criteria for Adverse Events (CTCAE v5.0 classification) (8). The CTCAE system is a product of the US National Cancer Institute (NCI) that uses a range of grades from 1 (mild) to 5 (death), specifying the specific conditions and symptoms that the patient must have to be classified in one or another category (**Supplementary Material**).

## 3 Results

At the time of the study (January 2023), 93 different OAAs had been approved by the EMA and were reviewed. Among them, 51.6% have recognized cardiotoxic AEs and 10.8% can cause alterations in lipid metabolism (**Table 1**).

**Table 1:** Cardiotoxic effect of oral antineoplastic agents.

QT Prolongation		
**Abiraterone - N/A** **Avapritinib - 2%** **Bosutinib - F** **Brigatinib - F** **Cabozantinib - N/A** **Capecitabine - R** **Ceritinib - F** **Crizotinib - 4%** **Cyclophosphamidec - N/A** **Dabrafenib - N/A**	Dasatinib - LF Enzalutamide - N/A Encorafenib - N/A Gilteritinib - 0.9% Lorlatinib - LF Lenalidomide - LF Nilotinib - F Osimertinib - 0.8% Pazopanib - LF	Pralsetinib - 5.1% Tepotinib - N/A Regorafenib - N/A Ribociclib - F Sorafenib - R Sunitinib - LF Tivozanib - LF Vandetanib - VF Vemurafenib - F
Decrease in left ventricular ejection fraction (LVEF)		
**Dabrafenib - F** **Dasatinib - LF** **Encorafenib - N/A** **Lapatinib - F**	Lorlatinib - LF Osimertinib - 3.2% Pazopanib - LF	Regorafenib - N/A Ripretinib - F Sunitinib - F
Hypertension		
**Abiraterone - VF** **Acalabrutinib - N/A** **Alpelisib - F** **Avapritinib - 33.3%** **Axitinib - N/A** **Azacitidine - F** **Bosutinib - F** **Cabozantinib - N/A** **Encorafenib - VF** **Enzalutamide - VF** **Etoposide - F** **Everolimus - F**	Ferdratinib - F Ibrutinib - 18% Imatinib - LF Lenalidomide - F Lenvatinib - 68.6% Lorlatinib - 13% Melphalan - 10% Nilotinib - F Nintedanib - F Niraparib - 19.3% Pazopanib - 41% Ponatinib - 2.9%	Pralsetinib - 33% Regorafenib - > 30% Relugolix - F Ripretinib - 19.4% Ruxolitinib - 15% Sorafenib - VF Sunitinib - VF Temozolamide - F Thalidomide - 10% Tivozanib - 47.6% Vandetanib - VF
Hypotension		
**Alectinib - N/A** **Azacitidine - F** **Busulfan - VF** **Capecitabine - LF**	Cyclophosphamide - N/A Dabrafenib - F Dasatinib - LF Gilteritinib - 17.2%	Mercaptopurine - N/A Methotrexate - R Pomalidomide - F Selinexor - F
Tachycardia		
**Abiraterone - 1.9%** ** Busulfan - VF** ** Capecitabine - LF** ** Cyclophosphamide - N/A** **Dasatinib - F** **Encorafenib - F** **Enzalutamide - N/A**	Erlotinib - N/A Etoposide - F Everolimus - LF Ibrutinib - LF Imatinib - LF Lenalidomide - F Mercaptopurine - N/A	Nilotinib - F Niraparib - VF Regorafenib - LF Ripretinib - F Osimertinib - 0.3% Tivozanib - F Vinorelbine - N/A
Bradycardia		
**Alectinib - VF** **Brigatinib - F** **Capecitabine - R**	Cyclophosphamide - N/A Crizotinib - 13% Dabrafenib - LF	Nilotinib - F Regorafenib - N/A Thalidomide - N/A
Thrombosis		
**Abemaciclib - F** ** Axitinib - F** ** Busulfan - VF** ** Cabozantinib - N/A** ** Capecitabine - LF** ** Cyclophosphamide - N/A** ** Dabrafenib - N/A** ** Dasatinib - LF** ** Encorafenib - F** **Erlotinib - N/A**	Everolimus - LF Fedratinib - N/A Imatinib - N/A Lenalidomide - VF Melphalan - N/A Methotrexate - R Nilotinib - LF Nintendanib - N/A Pazopanib - F Pomalidomide - F	Ponatinib - F Regorafenib - N/A Ruxolitinib - N/A Sorafenib - N/A Sunitinib - F Temozolamide - F Thalidomide - N/A Tivozanib - F Vinorelbine - N/A
Effects on lipid metabolism: Hypertriglyceridemia and/or hypercholesterolemia		
**Cabozantinib - N/A** **Capecitabine - LF** **Dasatinib - LF** **Everolimus - F**	Gilteritinib - N/A Ibrutinib - LF Lorlatinib - VF	Nilotinib - F Ruxolitinib - VF Vismodegib - N/A

VF, Very frequent (≥10%); F, Frequent (1-10%); LF, Less frequent (0.1-1%); R, Rare (0.01-0.1%); N/A, Not Available.

A total of 27 (29.0%) OAAs had specific recommendations regarding QT prolongation; 88.9% were listed in the SPC and 59.3% in Lexicomp^®^ (**Supplementary Material**). Recommendations on the management of QT prolongation described by the EMA and Lexicomp^®^ concurred in 80.0% of OAAs. In cases with different recommendations, no clinically relevant discrepancies were found between the two data sources. The main discrepancies were due to the lack of information from the EMA on acalabrutinib, dabrafenib, gilteritinib, regorafenib, relugolix, and vinorelbine, and from Lexicomp^®^ on alectinib, avapritinib, dabrafenib, cabozantinib, capecitabine, enzalutamide, encorafenib, lorlatinib lenalidomide, melphalan, methotrexate, imatinib, ponatinib, pomalidomide, pralsetinib, regorafenib, sunitinib, tepotinib, tivozanib, vandetanib, and vemurafenib in the analysis of some of the AEs studied. In general, EMA’s recommendations included more specific information for most of the drugs.

Regarding the decrease in LVEF, eight OAAs (9.7%) have reported this cardiotoxic effect, and four of these drugs, namely, encorafenib, lorlatinib, ripretinib, and sunitinib, have specific management recommendations (**Table 2**). It is worth noting that LVEF should be measured following international recommendations, preferably by echocardiogram and using 3D technology. In cases of an unclear echocardiography window, intravenous contrast can be administered or cardiac magnetic resonance as a gold standard can be performed (6).

**Table 2:** Specific recommendations concerning oral antineoplastic agents in case of a decrease in LVEF.

Drug	EMA	Lexicomp^®^
**Dabrafenib**	N/A	*Not indicated as an adverse effect*
**Dasatinib**	N/A	*Not indicated as an adverse effect*
**Encorafenib**	Monitor if administered in combination with binimetinib. Treatment with binimetinib and encorafenib should be discontinued if grade 3 or 4 decreases in LVEF, or an absolute decrease in LVEF ≥ 10% from baseline, and LVEF should be assessed every 2 weeks until recovery.	*Not indicated as an adverse effect*
**Osimertinib**	Cardiac monitoring, including baseline assessment of LVEF, during treatment.	Cardiac monitoring, including baseline assessment of LVEF, during treatment.
**Pazopanib**	Monitor.	N/A
**Regorafenib**	*Not indicated as an adverse effect*	N/A
**Ripretinib**	Grade 3 or 4: Permanently discontinue. Ejection fraction should be assessed by echocardiogram or multiple-gated acquisition (MUGA) scan prior to initiating ripretinib and during treatment, as clinically indicated.	Grade 3 or 4: Permanently discontinue.
**Sunitinib**	Monitor.	If LVEF <50% but >20% below baseline or below the lower limit of normal: Withhold until resolution to ≤ grade 1 or baseline, then resume at a reduced dose.
		If clinical manifestations of heart failure: Permanently discontinue sunitinib.

N/A, Not available information.

EMA, European Medicines Agency; LVEF, Left ventricular ejection fraction.

Almost half (49.5%) of currently approved OAAs can potentially alter blood pressure control; 34 (36.6%) of them have been reported to cause hypertension and 12 (12.9%) are related to hypotension. **Table 3** describes specific EMA and Lexicomp^®^ recommendations for each OAA in case of imbalances in blood pressure. First-line antihypertensive treatment should be ACE-I or ARB, as they also prevent LVEF decrease and are the first line of treatment for general hypertension. For cases with high levels (systolic HTN over 160 mmHg), the addition of a dihydropyrimidine calcium channel blocker can help to achieve faster control of HTN levels due to the combined effect of RASS inhibition and arterial vasodilation, counteracting endothelial toxicity of most of the TKIs (6). In most cases of persistent hypertension despite adequate medical treatment, the OAA should be temporarily interrupted and/or its dose reduced. Regarding the management of hypotension, we have only found recommendations for 2 of the 10 drugs involved: dabrafenib and dasatinib. The special case of abiraterone should be mentioned, while spironolactone is not recommended as it may interact with the androgen-synthesis pathway where abiraterone actuates (9). In addition, it is important for patients to actively monitor their blood pressure at home. Home blood pressure monitoring provides valuable insights about a patient’s blood pressure patterns throughout the day, which can help healthcare providers make informed treatment decisions. Regular monitoring empowers patients to actively participate in their hypertension management, leading to better control of blood pressure and improved health outcomes.

**Table 3:** Specific recommendations concerning oral antineoplastic agents in case of imbalances in blood pressure.

Hypertension		
Drug	EMA	Lexicomp^®^
**Abiraterone**	Caution in patients with underlying diseases.	Concomitant administration with corticosteroids reduces the incidence and severity of hypertension. Control BP and correct hypokalemia.
**Acalabrutinib**	*Not indicated as an adverse effect*	Grade ≥3: First and second occurrence: Interrupt acalabrutinib treatment; may resume at 100 mg every 12 hours after toxicity resolves to Grade 1 or baseline. Third occurrence: Interrupt acalabrutinib treatment; may resume with the dose reduced to 100 mg once daily after toxicity resolves to Grade 1 or baseline. Fourth occurrence: Discontinue acalabrutinib.
**Alpelisib**	Monitor before and during treatment.	N/A
**Avapritinib**	N/A	N/A
**Axitinib**	Monitor	Treat with standard antihypertensive therapy. Persistent hypertension: May require axitinib dose reduction. Severe, persistent (despite antihypertensives and dose reduction), or evidence of hypertensive crisis: Discontinue axitinib.
**Azacitidine**	N/A	N/A
**Bosutinib**	Interrupt and resume at a dose reduced by 100 mg after the toxicity has resolved. If clinically appropriate, re-escalation to the dose prior to the dose reduction taken once daily should be considered. Doses of less than 300 mg/day have been used in patients; however, efficacy has not been established.	Withhold bosutinib until resolved, then consider resuming with the daily dose reduced by 100 mg; may re-escalate the dose to the starting dose if clinically appropriate. Doses <300 mg daily have been used; however, efficacy has not been established.
**Cabozantinib**	Reduce dose in case of persistent hypertension despite the use of antihypertensive drugs. The administration should be discontinued in case of severe and persistent hypertension despite using antihypertensive therapy and dose reduction of cabozantinib.	Do not initiate cabozantinib in patients with uncontrolled hypertension. Grade 3: Withhold cabozantinib until hypertension is adequately controlled to ≤ grade 2, then resume at a reduced dose. Permanently discontinue cabozantinib for hypertension that cannot be controlled. Grade 4: Permanently discontinue cabozantinib. Hypertensive crisis: Permanently discontinue cabozantinib.
**Encorafenib**	N/A	*Not indicated as an adverse effect*
**Enzalutamide**	N/A	N/A
**Etoposide**	Appropriate supportive therapy.	N/A
**Everolimus**	N/A	N/A
**Ferdratinib**	N/A	N/A
**Ibrutinib**	Monitor whether antihypertensive medication should be instituted or adjusted.	May require initiation of antihypertensive therapy or adjustment of the existing antihypertensive regimen.
**Imatinib**	N/A	N/A
**Lenalidomide**	N/A	N/A
**Lorlatinib**	Grade 3: Withhold until hypertension has recovered to grade 1 or less (SBP < 140 mmHg and DBP < 90 mmHg), then resume lorlatinib at the same dose. If Grade 3 hypertension recurs: Withhold until recovery to grade 1 or less and resume at a reduced dose. If adequate hypertension control cannot be achieved with optimal medical management: Permanently discontinue lorlatinib.	Grade 3: Withhold lorlatinib until hypertension has recovered to ≤ grade 1 (SBP < 140 mmHg and DBP <90 mmHg), then resume at the same dose. If grade 3 hypertension recurs: Withhold lorlatinib until recovery to ≤ grade 1, then resume at a reduced dose. If hypertension cannot be adequately controlled with optimal medical management: permanently discontinue lorlatinib.
	Grade 4: Withhold until recovery to Grade 1 or less, and resume at a reduced dose or permanently discontinue. If Grade 4 hypertension recurs: permanently discontinue lorlatinib	Grade 4: Withhold lorlatinib until recovery to ≤ grade 1, then resume at a reduced dose or permanently discontinue lorlatinib. Recurrent grade 4 hypertension: Permanently discontinue lorlatinib.
**Melphalan**	N/A	N/A
**Nilotinib**	N/A	N/A
**Nintedanib**	N/A	N/A
**Niraparib**	Monitor before and during treatment.	If necessary, hypertension should be managed with antihypertensives and niraparib dose adjustment. Grade 3 or higher despite medical treatment: Withhold for a maximum of 28 days or until resolution; resume with the dose reduced. Grade 3 or higher treatment-related adverse reaction lasting >28 days at a dose of 100 mg once daily: Discontinue niraparib.
**Pazopanib**	Monitor	Grade 2 or 3: Reduce dose and initiate or adjust antihypertensive therapy. Permanently discontinue if hypertension remains at grade 3 despite dose reduction and adjustment of antihypertensive therapy.
		Grade 4 or hypertensive crisis: Permanently discontinue.
**Ponatinib**	Temporarily discontinue if hypertension is not medically controlled.	*Not indicated as an adverse effect*
**Pralsetinib**	Grade 3 Interrupt treatment if hypertension persists despite optimal antihypertensive therapy. Resume at a reduced dose when hypertension is controlled.	Grade 3 Initiate or optimize hypertensive therapy. Withhold pralsetinib for grade 3 hypertension that persists despite management with optimal antihypertensive therapy. Resume pralsetinib at a reduced dose when hypertension is controlled
	Grade 4 Permanently discontinue	Grade 4 Permanently discontinue
**Regorafenib**	Monitor. In cases of severe or persistent hypertension despite adequate medical treatment, treatment should be temporarily interrupted and/or its dose reduced at medical discretion. Dosage modifications should be made in steps of 40 mg. The minimum recommended daily dose is 80 mg. The maximum daily dose is 160 mg. Interruptions and/or dose reductions may be necessary depending on the safety and tolerability of each patient.	Associated with confusion, headache, chest pain, or dyspnea: May require urgent clinical intervention. Medically uncontrolled: Interrupt, reduce dose, or discontinue ponatinib. Significant worsening, labile, or treatment-resistant: Interrupt ponatinib and evaluate for renal artery stenosis
**Relugolix**	Hypertension should be treated and the benefit of continued treatment evaluated. If treatment is discontinued, Relugolix can be resumed if normal blood pressure values are achieved with antihypertensive treatment.	N/A
**Ripretinib**	Grade 3 If blood pressure is controlled to Grade ≤1 or baseline, resume at the same dose; otherwise, resume at a reduced dose. If Grade 3 hypertension recurs, withhold until symptoms have resolved and blood pressure is controlled. Resume at a reduced dose.	Grade 3 If blood pressure is controlled to Grade ≤1 or baseline, resume at the same dose; otherwise, resume at a reduced dose. If Grade 3 hypertension recurs, withhold until symptoms have resolved and blood pressure is controlled. Resume at a reduced dose.
	Grade 4 Permanently discontinue	Grade 4 Permanently discontinue
**Ruxolitinib**	N/A	Grade 3: Continue ruxolitinib at 1 dose level lower until recovery.
		Grade 4: Discontinue ruxolitinib
**Sorafenib**	Monitor regularly and treat if necessary. In case of severe or persistent hypertension, despite adequate antihypertensive treatment, permanent discontinuation of sorafenib should be considered.	Grade 2 symptomatic/persistent or Grade 2 symptomatic increase by >20 mm Hg (diastolic) or >140/90 mm Hg if previously within normal limits or grade 3: Interrupt until symptoms resolve and DBP is <90 mm Hg, then resume with the dose reduced by 1 dose level. If needed, reduce an additional dose level. If more than 2 dose reductions are necessary, permanently discontinue sorafenib.
		Grade 4: Permanently discontinue sorafenib
**Sunitinib**	Monitor regularly and treat if necessary. In case of severe or persistent hypertension, despite adequate antihypertensive treatment, permanent discontinuation of sorafenib should be considered. Treatment can be resumed once hypertension is adequately controlled.	N/A
**Temozolamide**	N/A	N/A
**Thalidomide**	N/A	N/A
**Tivozanib**	Monitor.	N/A
**Vandetanib**	Monitor. If hypertension cannot be controlled with clinical treatment, vandetanib should not be resumed until such BP is clinically controlled. Dose reduction may be necessary.	N/A
**Zanubrutinib**	N/A	N/A
Hypotension		
**Drug**	**EMA**	**Lexicomp^®^**
**Alectinib**	N/A	*Not indicated as an adverse effect*
**Azacitidine**	N/A	N/A
**Busulfan**	N/A	N/A
**Capecitabine**	N/A	N/A
**Cyclophosphamide**	N/A	N/A
**Dabrafenib**	Monitor and control with standard treatment.	*Not indicated as an adverse effect*
**Dasatinib**	Reduce dose.	N/A
**Gilteritinib**	N/A	N/A
**Mercaptopurine**	N/A	N/A
**Methotrexate**	N/A	N/A
**Pomalidomide**	N/A	*Not indicated as an adverse effect*
**Selinexor**	N/A	N/A

N/A, Not available information.

EMA, European Medicines Agency; BP, blood pressure; SBT, systolic blood pressure; DBT, diastolic blood pressure.

Tachycardia and/or bradycardia are associated with 22.58% and 8.6% of the evaluated drugs, respectively. Nilotinib and regorafenib are the only active substances that are known to cause both adverse effects (**Table 4**).

**Table 4:** Specific recommendations concerning oral antineoplastic agents in case of tachycardia and bradycardia.

Tachycardia		
Drug	EMA	Lexicomp^®^
**Dasatinib**	Monitor	N/A
**Ibrutinib**	Temporarily discontinue, and a full clinical benefit/risk assessment should be carried out before reinstitution of treatment.	N/A
**Regorafenib**	N/A	Discontinue until recovery, resume at the same dose, at a reduced dose, or discontinue depending on the severity and/or recurrence.
Bradycardia		
**Drug**	**EMA**	**Lexicomp^®^**
**Alectinib**	If treatment with a concomitant bradycardia-producing medication is identified and discontinued or its dose adjusted, treatment with alectinib can be restarted at the previous dose when the Grade of bradycardia is ≤ 1.	N/A
	Grade 3: Temporarily discontinue treatment until recovery of bradycardia to Grade ≤ 1 (asymptomatic) or until a heart rate ≥ 60 bpm.	
	Grade 4: Permanently discontinue treatment if no concomitant medication with known bradycardia-producing effect is identified.	
	Permanently discontinue treatment in case of recurrence.	
**Crizotinib**	Discontinue until recovery to Grade ≤ 1 or heart rate equal to or greater than 60.	If symptomatic bradycardia (not life-threatening) occurs, withhold treatment until recovery to asymptomatic bradycardia or to a heart rate of ≥60 beats/minute, evaluate concurrent medications, and potentially reduce crizotinib dose.
	If any concomitant medications contributing to bradycardia are identified and discontinued, or their dose is adjusted, restart at the previous dose when recovery to Grade ≤ 1 or a heart rate equal to or greater than 60.	Avoid concurrent use with other agents known to cause bradycardia.
	If life-threatening consequences, emergency intervention is indicated: Discontinue permanently if no concomitant medications contributing to bradycardia are identified. If any concomitant medications contributing to bradycardia are identified and discontinued, or its dose is adjusted, restart with 250 mg daily when recovery to Grade ≤ 1 or to a heart rate equal to or greater than 60, and monitor the patient frequently.	Permanently discontinue for life-threatening bradycardia due to crizotinib; if life-threatening bradycardia occurs and concurrent medications associated with bradycardia can be discontinued or dose adjusted, restart crizotinib at a reduced dose (with frequent monitoring).
**Thalidomide**	The dosing interval may need to be increased, reduced, or the treatment discontinued, depending on the degree of toxicity according to the CTCAE scale.	N/A

N/A, Not available information; EMA, European Medicines Agency.

Regarding thrombosis, 30 (32.3%) of the drugs analyzed included the appearance of a thrombus as a possible AE (**Table 5**). Specific management recommendations are issued in seven cases and often indicate OAA discontinuation in case of occurrence. For treatments with melphalan, lenalidomide, pomalidomide, or thalidomide, prophylactic antithrombotic treatment is recommended.

**Table 5:** Specific recommendations concerning oral antineoplastic agents in case of thrombosis.

Drug	EMA	Lexicomp^®^
**Abemaciclib**	Monitor signs and symptoms.	Grade 1 or 2: No dosage modification is required.
		Grade 3 or 4: Withhold and manage as clinically indicated. Resume when clinically stable.
**Axitinib**	N/A	N/A
**Busulfan**	N/A	N/A
**Cabozantinib**	Discontinue if any thromboembolic complication.	Permanently discontinue.
**Capecitabine**	N/A	N/A
**Cyclophosphamide**	N/A	N/A
**Dabrafenib**	Discontinue.	Uncomplicated: No dosage modification is necessary.
**Dasatinib**	N/A	N/A
**Encorafenib**	N/A	*Not indicated as an adverse effect*
**Erlotinib**	N/A	N/A
**Everolimus**	N/A	N/A
**Ferdratinib**	Prior to initiating or continuing therapy, consider risk vs benefit, particularly in patients with cardiovascular factors. Patients should be re-evaluated periodically during treatment to assess for changes in VTE risk.	Monitor.
**Imatinib**	N/A	*Not indicated as an adverse effect*
**Lenalidomide**	Thromboprophylaxis is recommended.	Thromboprophylaxis is recommended.
**Melphalan**	Thromboprophylaxis is recommended.	*Not indicated as an adverse effect*
**Methotrexate**	N/A	*Not indicated as an adverse effect*
**Nilotinib**	N/A	N/A
**Nintedanib**	Discontinue if life-threatening venous thromboembolic reactions.	Monitor
**Pazopanib**	A treatment decision should be based on individual patient benefit/risk assessment.	Any grade: Permanently discontinue.
		Grade 3: Withhold. Resume pazopanib at the same dose if managed with appropriate therapy for at least 1 week.
		Grade 4: Permanently discontinue.
**Pomalidomide**	Thromboprophylaxis is recommended.	Thromboprophylaxis is recommended.
**Ponatinib**	N/A	*Not indicated as an adverse effect*
**Regorafenib**	N/A	N/A
**Ruxolitinib**	N/A	N/A
**Sorafenib**	N/A	N/A
**Sunitinib**	N/A	*Not indicated as an adverse effect*
**Temozolamide**	N/A	*Not indicated as an adverse effect*
**Thalidomide**	Thromboprophylaxis is recommended.	Consider thromboprophylaxis based on an assessment of individual patient’s underlying risk factors.
**Tivozanib**	N/A	N/A
**Vinorelbine**	*Not indicated as an adverse effect*	N/A

N/A, Not available information; EMA, European Medicines Agency.

A total of 10 OAAs have been associated with hypertriglyceridemia and hypercholesterolemia; we found that 3 (everolimus, lorlatinib, and nilotinib) out of 10 have a specific dose adjustment management (**Table 6**).

**Table 6:** Specific recommendations concerning oral antineoplastic agents of changes in the lipidic metabolism.

Drug	Alteration	EMA	Lexicomp^®^
**Cabozantinib**	Dyslipidemia	N/A	N/A
**Capecitabine**	Hypertriglycemia	N/A	N/A
**Dasatinib**	Hypercholesterolemia	N/A	N/A
**Everolimus**	Hypertriglycemia and dyslipidemia	Grade 2: No dose adjustment is required.	N/A
		Grade 3: Temporary interruption until improvement. Restart at the dose of 5 mg per day.	Grade 3: Temporary interruption until improvement. Restart at 50% of the previous dose or alternate day dosing if the reduced dose is less than the lowest potency available.
		Grade 4: Permanently discontinue treatment.	Grade 4: Permanently discontinue treatment.
**Gilteritinib**	Hypertriglycemia	*Not indicated as an adverse effect*	N/A
**Ibrutinib**	Hypertriglycemia	N/A	N/A
**Lorlatinib**	Hypercholesterolemia and Hypertriglyceridemia	If cholesterol is between ULN and 400 mg/dL or triglycerides between 150 and 300 mg/dL: Introduce or modify lipid-lowering therapy. Continue lorlatinib at the same dose.	Grade 4 hypercholesterolemia or grade 4 hypertriglyceridemia: Withhold until recovery to ≤ grade 2 and then resume lorlatinib at the same dose. If severe hypercholesterolemia and/or hypertriglyceridemia recurs, resume at a reduced dose. Hyperlipidemia may require initiation (or increased doses) of lipid-lowering agents.
		If cholesterol between 401 and 500 mg/dL or triglycerides between 501 and 1.000 mg/dL: Introduce the use of lipid-lowering therapy; if currently on lipid-lowering therapy, increase the dose of this therapy or change to a new lipid-lowering therapy. Continue lorlatinib at the same dose without interruption.	
		If cholesterol is over 500 mg/dL or over or triglycerides over 1,000 mg/dL: Introduce the use of lipid-lowering therapy or increase the dose of this therapy or change to a new lipid-lowering therapy. Withhold lorlatinib until the recovery of hypercholesterolemia and/or hypertriglyceridemia to moderate or mild severity. Re-challenge at the same lorlatinib dose while maximizing lipid-lowering therapy.	
**Nilotinib**	Hypercholesterolemia	Determine lipid profiles before starting treatment and evaluate them every 3 months. Consult interactions if an HMG-CoA reductase inhibitor (lipid-lowering agent) is needed.	N/A
**Ruxolitinib**	Hypertriglycemia	N/A	N/A
**Vismodegib**	Hypertriglycemia	N/A	N/A

N/A, Not available information.

EMA, European Medicines Agency; ULN, upper limit of normal.

## 4 Discussion

To the best of our knowledge, this is the first guide to summarize the updated recommendations concerning the use of OAAs with their cardiotoxic effects and posology adjustments according to the EMA and Lexicomp^®^.

The fast inclusion in the clinical practice of OAAs, their significant adverse events, and the scarce experience with some groups of patients that were not included in clinical trials make it extremely important that careful management of OAAs is implemented. The mechanism and frequency of cardiotoxicity brought on by targeted molecular therapy are mainly unclear and probably underestimated. Healthcare professionals play a crucial role in patient education, assisting in identifying and managing AEs and drug interactions. Recently, the European Society of Cardiology (ESC) issued its first guideline, providing guidance on the definitions, diagnosis, treatment, and prevention of cancer therapy-related CV toxicity, and the management of CV disease caused directly or indirectly by cancer (6). However, this guideline does not detail specific recommendations for each of the drugs or dose adjustments. Therefore, we suggest that our review complements the recommendations provided by the ESC.

Our review presents a comprehensive guide of available recommendations concerning the dose and posology of OAAs when cardiotoxic events appear. We aimed to compile these recommendations to help clinicians to decide when to change the dose, end treatment, or take another action when a cardiac AE is detected. Considering the limitations of the information used to establish the safety profile of drugs during the premarketing period, there is a need for continuous safety surveillance after approval. As many of these therapeutic agents are approved through expedited procedures, the number of SPC changes in the postmarketing phase due to safety issues has increased. Likewise, it should be noted that specific information on dose adjustment recommendations in cases of adverse effects is reduced so that, in many cases, the recommendations are general for each side effect and not specific for each drug.

### 4.1 QT prolongation

Tyrosine kinase inhibitors are the therapeutic class most frequently associated with cardiac toxicities such as QT prolongation, left ventricular dysfunction, or arterial hypertension. QT prolongation is a severe cardiotoxic effect, which can lead to ventricular tachycardia (*Torsades de Pointes*) (10). Although this AE is rare, it is potentially life-threatening, and there is currently not much data on the prevalence of Torsade de Pointes in patients with cancer. We have identified that 29.0% of the OAAs analyzed can prolong the QT interval. A recent review that focused on QT prolongation graded 205 anticancer drugs as low, moderate, and high risk of QT prolongation. The authors identified eight drugs with a high risk of QT prolongation: arsenic trioxide, imatinib, ivosidenib, nilotinib, ribociclib, thiopental, toremifene, and vandetanib (11).

Several risk factors may increase the risk of QT prolongation. Among the non-cardiac risk factors are being from the female sex and hypothyroidism. Among the cardiac risk factors are congenital long QT syndrome, left ventricular dysfunction, and myocardial ischemia. In addition to QT prolongation, risk factors also increase the risk of concomitant treatments (antidepressants, antiemetics, antibiotics, antipsychotics, anti-fungal, antihistamines, and methadone) and AEs associated with cancer therapy (nausea and vomiting, dehydration followed by electrolyte imbalances such as hypokalemia, hypomagnesemia, and hypocalcemia, and of other adverse effects such as kidney failure, liver dysfunction, and poorly controlled diabetes) (12). Before starting a new OAA, these risk factors should be evaluated and corrected as possible, specifically potassium, calcium, and magnesium levels. Besides, patients with cancer often are polymedicated; thus, it is essential to check the possible drug-drug interactions between OAAs and cytochrome P450 3A4 inhibitors (amiodarone, digoxin, antihistamines, atorvastatin, carbamazepine, corticosteroids, etc.) since they can increase the plasma concentrations of many of the antiangiogenic protein kinase inhibitors. A dose reduction of the OAA may be necessary in these cases. That is the reason why ESC guidelines recommend avoiding heterogeneity in the method of corrected-QT measurement, defining a clear threshold of 500 msec as risky, and ruling out other causes of QT prolongation as ionic imbalances or other QT-prolonging drugs frequently prescribed in cancer patients (6). Otherwise, a QT prolongation can be interpreted as a concern and lead to an early withdrawal of a potentially beneficial antitumoral drug. The multidisciplinary management of cardiotoxicity in cardio-oncology units is mandatory to avoid such an error and should be generalized.

### 4.2 LVEF decrease

Left ventricular dysfunction and heart failure may occur due to mitochondrial damage, alterations in cardiac energy balance, and contractile protein dysfunction (13). The inhibition of platelet-derived growth factor receptor (PDGFR) and other tyrosine kinase receptors in cardiomyocytes, which determines their functioning and survival, disrupts the typical response of the myocyte to hypertensive stress (14). We have identified that 9.7% of the analyzed OAAs can decrease LVEF.

Various studies demonstrated heart failure with reduced ejection fraction or other cardiomyopathies induced by OAAs, such as encorafenib, lorlatinib, osimertinib, or sunitinib (15–17). Patel SR et al. observed, in a series of cases, that cardiotoxicity from osimertinib was reversible. They hypothesized that dose reduction may be a potential solution to prevent initial or recurrent cardiotoxicity (18). The initiation of heart failure drugs is recommended, and these patients should be referred to cardio-oncology for further studies and follow-up (19). Rechallenge of the drug may be considered under strict surveillance after a multidisciplinary discussion on the risk/benefit ratio (6).

### 4.3 Hypertension and hypotension

Hypertension is one of the most common comorbidities in cancer survivors and patients with active cancer. Its incidence varies according to age, history of hypertension, cancer type, and treatment. A retrospective large cohort study was conducted to estimate the incidence rates of new-onset hypertension in adult cancer patients. New-onset hypertension was observed in approximately one-third of the 25,090 patients with various types of cancer. The incidence rates of severe and crisis-level hypertension were highest in patients with gastric and ovarian cancer (20). Across all cancers, chemotherapy exposure was associated with a 2- to 3.5-fold increase in the risk of any grade of hypertension compared with periods without chemotherapy; higher hypertension levels showed more significant variability in relative risks by type and line of treatment but indicated an overall increase associated with chemotherapy exposure (21).

The mechanism by which OAAs induce hypertension is not entirely understood but can be directly related to the inhibition of vascular endothelial growth factor (VEGF) signaling via tyrosine kinase (22). The VEGF signaling pathway is also present in normal vascular endothelium, playing a physiological role in its function and in nitric oxide synthesis, thus its block compromises vasodilation. The pro-hypertensive mechanism of action is a class effect and is directly related to its antitumor action, so the increase in blood pressure, paradoxically, may be a marker of the effectiveness of oncologic treatment (23). Fortunately, blood pressure control with antihypertensive drugs does not seem to affect the effectiveness of anticancer treatment. The hypertensive effect of these agents is reversible, so a decrease in dose or temporary discontinuation of treatment can be used to control hypertension. This applies especially to protein kinase inhibitors, as they are administered daily orally for prolonged periods and can be restarted, or the dose adjusted once hypertension has been controlled with appropriate medication. Strict blood pressure control reduces the risk of heart failure and atrial fibrillation and avoids the need to interrupt effective anticancer treatment (24). According to our study, 36.6% of OAAs have hypertensive potential. Specifically, angiogenesis inhibitor drugs (VEGF pathway inhibitors) have a class effect on blood pressure and show the most significant problems. Introducing these drugs highlighted the importance of hypertension during cancer treatment since, as a group, they can induce it with a frequency that has been variably estimated to be between 17% and 80% of cases in different studies (25).

### 4.4 Lipid imbalance

The pathogenesis of hypertriglyceridemia associated with mTOR agent use, such as everolimus, is poorly understood but may be related to the reduced degradation of apolipoprotein B100 (26). Apolipoprotein B100 is formed in the liver and is essential to the assembly of very low-density lipoproteins. Additionally, everolimus may lower levels of lipoprotein lipase activity and increase free fatty acid levels, which can contribute to dyslipidemia (27). Specific recommendations for controlling cardiovascular risk factors in patients with cancer have been agreed upon by Spanish societies of cardiology, oncology, hematology, radiation oncology, and general practitioners and can serve as a guide for the management of this frequent adverse effect (3).

Screening for lipid levels is recommended for individuals without cardiovascular disease (CVD), especially for those with risk factors such as hypertension, family history of premature CVD, diabetes, and smoking. Lifestyle interventions, including smoking cessation, a Mediterranean diet, and exercise, should be discussed with all patients. For secondary prevention patients, high-intensity statin therapy is strongly encouraged, while for primary prevention patients, the decision to initiate moderate- or high-intensity statins should be based on individual CVD risk. Statin therapy is generally not recommended for primary prevention patients over 75 years, but exceptions can be made based on individual patient factors. Patients who cannot tolerate specific statin regimens may be offered lower-intensity options or alternate dosing schedules. Non-statin lipid-lowering drugs are not recommended as first-line monotherapy in primary prevention. Adherence to statin therapy should be reinforced, and monitoring of CK and ALT levels should be reserved for symptomatic or higher-risk patients as determined by the attending clinician (28).

### 4.5 Thrombosis

Regarding the thrombotic effect, some studies emphasized the importance of the ribosomal S6 kinase family, as it determines cardiomyocyte survival by inhibiting the phosphorylation of apoptosis-activating factors (2). By interfering with this molecular pathway, KIs may promote cardiac damage. The inhibition of the KIT and RAF1 pathways leads to vascular stem cell damage and endothelial dysfunction. Endothelial cell apoptosis and exposure to subendothelial collagen initiate the coagulation process, leading to thromboembolic episodes. Approximately 20% of patients with venous thromboembolism (VTE) have current cancer, according to extensive population-based studies and disease registry surveys. In these patients, VTE is linked to decreased survival and negatively affects the quality of life (29). In our review, we identified that 32.3% of OAAs may increase the risk of thrombosis. We also observed that patients with cancer are especially at risk of developing thrombosis.

In terms of study limitations, we have not found in the sources consulted any recommendations for the management of some OAAs, despite their cardiotoxicity. Therefore, the application of this guide would help to identify them as cardiotoxic drugs, but not help in their management. Second, we have focused this review on OAAs, but it should be taken into account that other cancer treatments, such as parenteral chemotherapy, immunotherapy, or monoclonal antibodies, can also produce cardiotoxic effects. In addition, it should be noted that continued research and approval of new OAAs are being developed. Finally, data were obtained from EMA and Lexicomp; thus, in cases with limited information, other sources, such as data from clinical trials, can be consulted to supplement the available information. Having the EMA and Lexicomp recommendations in the same Table could confuse some healthcare professionals as to which recommendation to follow. However, the objective is to have more information available, which can be useful in different situations. For example, a European professional could follow the EMA recommendations, or if another professional has doubts, it may be useful to consult both sources.

In conclusion, more than half of the OAAs can produce cardiotoxic effects, with the most frequent being blood pressure alteration and QT interval prolongation with a non-depreciable incidence of LV dysfunction or thrombosis. Before starting the treatment, it is necessary to stratify baseline cardiovascular risk, plan a surveillance schedule, and consider referral to cardio-oncology units, especially in high-risk patients such as those with pre-existing bradycardia, those taking anti-arrhythmic medicines or other drugs known to prolong QT intervals, and patients with previous heart disease or developed cardiotoxicity.

We suggest that this review can serve as a reference guide for the professionals involved in managing patients with cancer since it provides an easy way to identify the cardiotoxic effects of OAAs and the need for dosage adjustments.

## Data availability statement

The original contributions presented in the study are included in the article/**Supplementary Material**. Further inquiries can be directed to the corresponding author.

## Author contributions

VE-V, RC-B, and CV-B defined the research question and objectives. VE-V, RC-B, CV-B, JR-H, SP-R, and EZ-N defined the methodology of the study. ER-R, PR-B, VE-V, XG-G, and SI-G conducted a search for information for the preparation of the guide. VE-V, AH, and MS were responsible for the research activity plan and its execution. ER-R and VE-V wrote the first draft. All authors contributed to the article and approved the submitted version.
