# Supplementary material for: Medication guide for dose adjustment and management of cardiotoxicity and lipid metabolic adverse events of oral antineoplastic therapy
Source: Front Oncol. 2023 Aug 25;13:1220305. doi: 10.3389/fonc.2023.1220305 (PMC10485602; doi:10.3389/fonc.2023.1220305)
Supplement: Supplementary file 1 [file Table_1.docx]

**Supplemental material. Specific recommendations concerning oral antineoplastic agents in case of QT prolongation.**

| **Drug** | **EMA** | **Lexicomp®** |
| --- | --- | --- |
| **Abiraterone** | Monitor other medications that increase QT interval | Monitor patients who develop hypokalemia. |
| **Avapritinib** | Monitor and use with caution in patients with known QT interval prolongation or at risk.  If concomitant use with a moderate CYP3A inhibitor cannot be avoided, the starting dose of avapritinib must be reduced from 200 mg to 50 mg orally once daily. | *Not indicated as an adverse effect* |
| **Bosutinib** | Monitor. Correct hypokalemia and hypomagnesemia. | Withhold bosutinib until resolved, then consider resuming with the daily dose reduced by 100 mg. May re-escalate the dose to the starting dose if clinically appropriate. |
| **Cyclophosphamide** | N/A | N/A |
| **Cabozantinib** | Monitor ECG and electrolytes (serum Ca ~ 9.0 mmol/L, K ~ 4.0 mmol/L, and Mg ~ 2 mmol/L) during treatment. | *Not indicated as an adverse effect* |
| **Capecitabine** | N/A | *Not indicated as an adverse effect* |
| **Crizotinib** | Grade 3 QT prolongation: Discontinue until recovery to Grade ≤ 1. Check electrolytes (serum Ca ~ 9.0 mmol/L, K ~ 4.0 mmol/L, and Mg ~ 2 mmol/L) and correct them if necessary. Then restart with the next lower dose. | QT >500 msec on at least 2 separate ECGs: Withhold crizotinib until recovery to QT <481 msec or baseline, then resume at the next lower dose. |
|  | Grade 4 QT prolongation: Suspend permanently. | QT >500 msec or ≥60 msec with torsades de pointes or polymorphic ventricular tachycardia or signs/symptoms of serious arrhythmia: Permanently discontinue crizotinib. |
|  | QT ≥ 60 msec but < 500 msec: Discontinue and consult a cardiologist. | Symptomatic bradycardia (heart rate <60 beats/minute): Withhold until recovery. |
|  | QT ≥ 500: Consult a cardiologist. | Life-threatening bradycardia with urgent intervention: Withhold until recovery to asymptomatic bradycardia or to a heart rate of ≥60 beats/minute and evaluate concomitant medications. If contributing concomitant medication is identified and discontinued (or is dose adjusted), then (upon recovery) resume crizotinib at the 2nd dose reduction with frequent monitoring. If no contributing concomitant medication is identified, permanently discontinue crizotinib. |
| **Dabrafenib** | *Not indicated as an adverse effect* | Monitor |
| **Dasatinib** | Caution in patients with congenital prolonged QT syndrome or taking drugs that induce QT prolongation and patients on treatment with high cumulative doses of anthracyclines. Hypokalemia or hypomagnesemia should be corrected prior to dasatinib administration. | Monitor. |
| **Enzalutamide** | Caution with patients taking concomitant medication that may cause QT interval prolongation. | *Not indicated as an adverse effect* |
| **Encorafenib** | QT >500 msec with an increase >60 msec concerning the pretreatment value: Treatment should be permanently discontinued. | QT >500 msec and >60 msec increase from baseline: Permanently discontinue encorafenib. |
|  | QT>500 msec with a variation ≤60 ms from pretreatment value: Treatment should be discontinued. Treatment with encorafenib at a reduced dose should be resumed when the QT is ≤500 msec. Encorafenib should be discontinued if more than one relapse occurs. | QT >500 msec and ≤60 msec increase from baseline: Withhold encorafenib until QT is ≤500 msec; then resume at a reduced dose. If more than 1 recurrence, permanently discontinue encorafenib. |
| **Gilteritinib** | ECG should be performed before initiation of treatment (day 8 and 15 of cycle 1) and before the start of the next three subsequent months of treatment. Caution with patients with a relevant cardiac history, hypokalemia, or hypomagnesemia. | QT interval >500 msec: Interrupt gilteritinib; when QT interval returns to within 30 msec of baseline or ≤480 msec, resume therapy at a reduced dose of 80 mg once daily. |
|  | QT >500 msec: Discontinue. If gilteritinib is re-introduced at a reduced dose, ECG should be performed after 15 days of dosing and before starting the next three months of treatment. | QT interval increased by >30 msec on ECG on day 8 of cycle 1: Confirm with a repeat ECG on day 9. If confirmed, consider dose reduction to 80 mg once daily. |
| **Lorlatinib** | N/A | *Not indicated as an adverse effect* |
| **Lenalidomide** | N/A | *Not indicated as an adverse effect* |
| **Nilotinib** | Monitor ECG, hypokalemia, and hypomagnesemia. | QT prolongation >480 msec: Withhold nilotinib and monitor and correct K and Mg levels; review concurrent medications. If QT returns to <450 msec and to within 20 msec of baseline within 2 weeks: Resume at prior dose. |
|  |  | QT returns to 450 to 480 msec after 2 weeks: Reduce dose to 230 mg/m2 once daily. |
|  |  | QT returns to >480 msec after dosage reduction to 230 mg/m^2^ once daily: Discontinue. Repeat ECG ~7 days after any dosage adjustment. |
| **Osimertinib** | QT interval greater than 500 msec in at least 2 independent ECGs: Discontinue until QT interval is less than 481 msec, or recovery to baseline if baseline QT is equal to or greater than 481 msec, then resume at a reduced dose (40 mg). | QT interval >500 msec on at least 2 separate ECGs: Withhold treatment until QT interval is <481 msec or recovers to baseline (if baseline QT ≥481 msec) and then resume at a dose of 40 mg once daily. |
|  |  | QT interval prolongation with signs/symptoms of life-threatening arrhythmia: Permanently discontinue. |
|  |  | Symptomatic heart failure: Permanently discontinue osimertinib. |
| **Pazopanib** | Monitor ECG, hypokalemia, and hypomagnesemia. | N/A |
| **Pralsetinib** | Grade 3 Interrupt treatment for QTc intervals >500 ms until QTc interval returns to <470 ms. Resume at the same dose if risk factors that cause QT prolongation are identified and corrected. Resume treatment at a reduced dose if other risk factors that cause QT prolongation are not identified. | *Not indicated as an adverse effect* |
|  | Grade 4 Permanently discontinue if has life-threatening arrhythmia. |  |
| **Regorafenib** | *Not indicated as an adverse effect* | N/A |
| **Ribociclib** | QT >480 msec: Dose should be discontinued | QT >480 msec: Interrupt treatment; when QT resolves to <481 msec; resume at the next lower dose level. |
|  | QT prolongation resolves to <481 msec: restart treatment at the next lower dose level. | QT >500 msec: Interrupt treatment for QT >500 msec; if QT resolves to <481 msec, may resume at the next lower dose level. |
|  | QT prolongation resolves to <481 msec: restart treatment at the next lower dose level. | QT interval prolongation is either >500 msec or >60 msec: Permanently discontinue. |
|  | QT ≥481 msec recurs: discontinue dosing until QT ≥481 msec is resolved. QT resolves to <481 msec and restart at the next lower dose level. |  |
| **Sorafenib** | Monitor ECG, hypokalemia, and hypomagnesemia. | QT interval >500 msec or ≥60 msec increase from baseline: Interrupt treatment and correct electrolyte abnormality. Use medical judgment before restarting. |
| **Sunitinib** | N/A | Monitor |
| **Tepotinib** | Monitor ECG and electrolytes (serum Ca ~ 9.0 mmol/L, K ~ 4.0 mmol/L, and Mg ~ 2 mmol/L). | *Not indicated as an adverse effect* |
| **Tivozanib** | Monitor ECG, hypokalemia, and hypomagnesemia. | *Not indicated as an adverse effect* |
| **Vandetanib** | Dosing should be discontinued temporarily and resumed at a reduced dose when toxicity has resolved or improved to CTCAE grade 1. The daily dose of 300 mg can be reduced to 200 mg and then to 100 mg if necessary. The patient should be monitored appropriately. | *Not indicated as an adverse effect* |
| **Vemurafenib** | QT > 500 msec and changes > 60 msec from pretreatment values: Permanently discontinue. | *Not indicated as an adverse effect* |
|  | 1st occurrence of QT>500 msec: Temporarily interrupt treatment until QT decreases below 500 msec. Resume dosing at 720 mg twice daily (or 480 mg twice daily if the dose has already been lowered). |  |
|  | 2nd occurrence of QT > 500 msec: Temporarily discontinue treatment until QT decreases below 500 msec. Resume dosing at 480 mg twice daily (or discontinue permanently if the dose has already been decreased to 480 mg twice daily). |  |
|  | 3rd occurrence of QT > 500 msec: Discontinue permanently. |  |

N/A: Not available information.

ECG: electrocardiogram; EMA: European Medicines Agency.
